# Two-Exon Skipping within *MLPH* Is Associated with Coat Color Dilution in Rabbits

**DOI:** 10.1371/journal.pone.0084525

**Published:** 2013-12-20

**Authors:** Stefanie Lehner, Marion Gähle, Claudia Dierks, Ricarda Stelter, Jonathan Gerber, Ralph Brehm, Ottmar Distl

**Affiliations:** 1 Institute for Animal Breeding and Genetics, University of Veterinary Medicine Hannover, Hannover, Germany; 2 Institute for Anatomy, University of Veterinary Medicine Hannover, Hannover, Germany; 3 Clinic for Pets, Reptiles and Pet and Feral Birds, University of Veterinary Medicine Hannover, Hannover, Germany; University of Sydney, Australia

## Abstract

Coat color dilution turns black coat color to blue and red color to cream and is a characteristic in many mammalian species. Matings among Netherland Dwarf, Loh, and Lionhead Dwarf rabbits over two generations gave evidence for a monogenic autosomal recessive inheritance of coat colour dilution. Histological analyses showed non-uniformly distributed, large, agglomerating melanin granules in the hair bulbs of coat color diluted rabbits. We sequenced the cDNA of *MLPH* in two dilute and one black rabbit for polymorphism detection. In both color diluted rabbits, skipping of exons 3 and 4 was present resulting in altered amino acids at p.QGL[37-39]QWA and a premature stop codon at p.K40*. Sequencing of genomic DNA revealed a c.111-5C>A splice acceptor mutation within the polypyrimidine tract of intron 2 within *MLPH*. This mutation presumably causes skipping of exons 3 and 4. In 14/15 dilute rabbits, the c.111-5C>A mutation was homozygous and in a further dilute rabbit, heterozygous and in combination with a homozygous frame shift mutation within exon 6 (c.585delG). In conclusion, our results demonstrated a colour dilution associated *MLPH* splice variant causing a strongly truncated protein (p.Q37QfsX4). An involvement of further *MLPH*-associated mutations needs further investigations.

## Introduction

Coat color dilution in rabbits affects eumelanin as well as pheomelanin. A dilution of eumelanin in black and brown coat color leads to blue and cream-brown, respectively, while a dilution of pheomelanin in yellow coat color leads to cream-yellow [1]. The rabbit dilution allele (d) is a monogenic, autosomal recessive trait [1]. Besides the rabbit, color dilution of the same phenotype is known and often favoured by selective breeding in many different animal species including cats [2], chicken [3], quails [4], mice [5], foxes [6], and minks [7]. Dilution of the coat color is also seen in dogs where color-dilution can be accompanied by alopecia [8-10]. This defect causes poor quality of the hair coat and hair re-growth to the point of progressive and extensive hair loss as well as comedo formation [9]. It was not reported for the dilution phenotype in any of the other species mentioned above. In humans, Griscelli syndromes are characterized by pigmentary dilution due to large agglutinations of pigment in the hair shafts and accumulation of melanosomes in melanocytes [11]. Similar histological findings including uneven granule-distribution and pigment agglutinations especially around the nucleus were found in dilute cats [2], foxes [6] and mice [5,12]. In diluted dogs, hair shafts, follicles, and bulbs showed a massive, abnormal clumping of melanin in the epidermis, dermis, and hair follicles [9,10,13]. For the coordination of melanosome capture, transport, and distribution, a tripartite protein complex of Rab27A, melanophilin, and Myosin Va was shown to be essential [14]. In this complex, Rab27A binds to melanosomes and captures melanophilin, which subsequently captures myosin Va. Myosin Va again binds to actin filaments and thus facilitates melanosome transport [14-17]. In human, mutations within any of the genes *RAB27A* (*RAB27A*, *member RAS oncogene family*), *MLPH* (*melanophilin*), and *MYO5A* (*myosin VA*) encoding for the proteins of the tripartite complex can cause Griscelli syndromes. However, dilution of the hair is commonly accompanied by further symptoms depending on the defective gene [18]. Griscelli syndrome type 1 (GS1) is caused by mutations of *MYO5A* and hypomelanosis is accompanied by neurologic deficits [19], while Griscelli syndrome type 2 (GS2) is caused by *RAB27A* mutations and patients show immune impairment in combination with the hair color dilution [20]. Griscelli syndrome type 3 (GS3) is caused by mutations within *MLPH* or *MYO5A* [21] and only *MLPH* defects are usually not accompanied by severe clinical diseases [22]. The human Griscelli phenotypes GS1, GS2, and GS3 correspond to the dilute, ashen, and leaden phenotypes in the mouse, respectively [23] and the human Griscelli phenotype GS1 corresponds to the lavender foal syndrome in the horse [24].

In this study, we analysed the dilute coat color in rabbits. *MLPH* was chosen as the most likely candidate gene due to histological analyses and phenotypic examination. We sequenced the cDNA of *MLPH* in black and dilute rabbits and performed mutation screening for the dilution phenotype.

## Results

### Phenotypes and Histological Analyses

**Figure 1:**
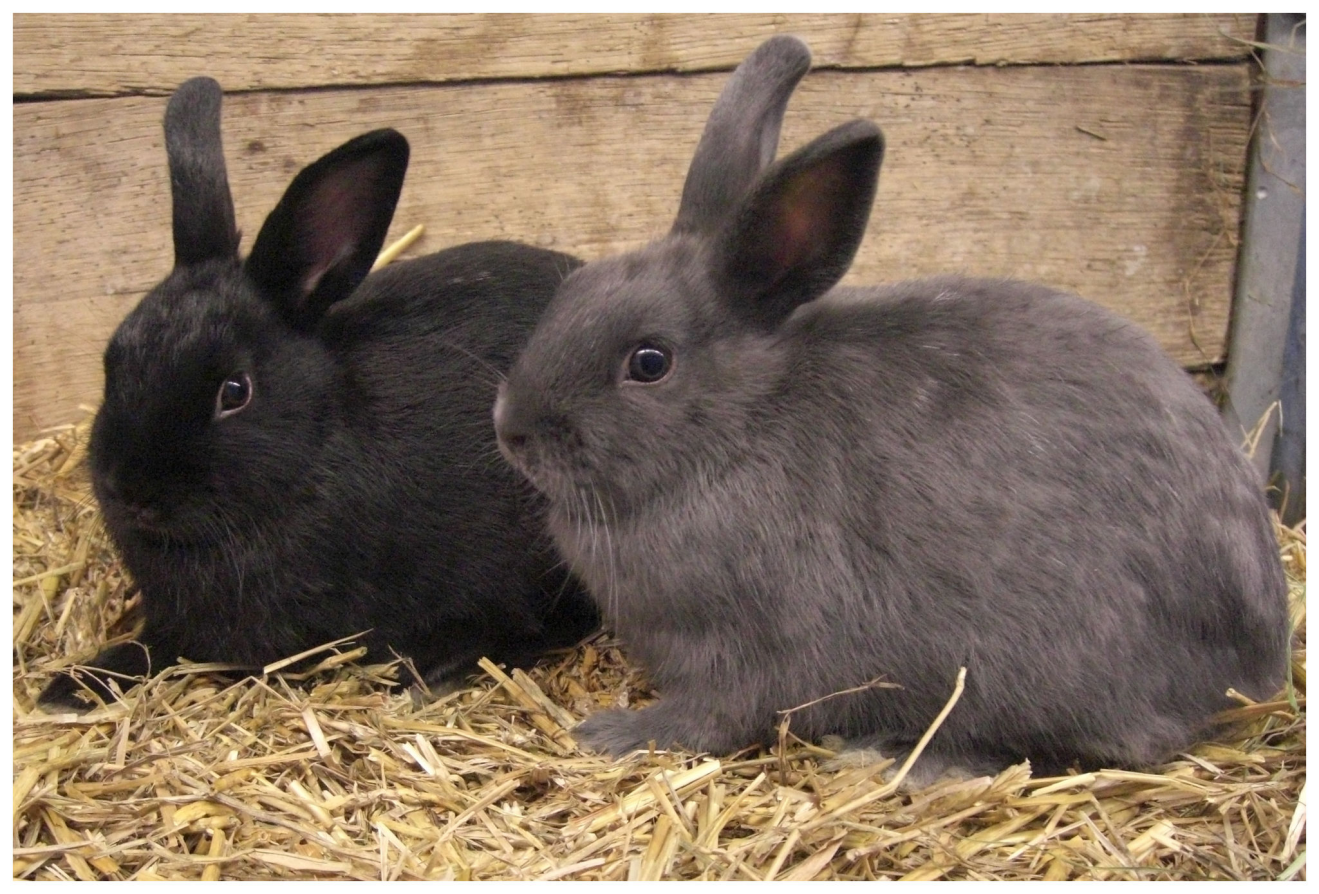
Phenotypic appearance of black (wildtype) and blue (dilute) rabbits.

In total 23 Netherland Dwarf, Loh, and Lionhead Dwarf rabbits and their crosses of our breeding trial, including six individuals showing coat color dilution, were clinically examined for defects accompanying the dilute color. No abnormal clinical findings were detected associated with this phenotype, neither serious clinical defects nor less severe signs like color dilution alopecia or comedo formations. The oldest rabbit showing the dilution phenotype was more than three years of age at examination. The phenotypes of a black and a blue colored rabbit are shown in Figure 1.

**Figure 2:**
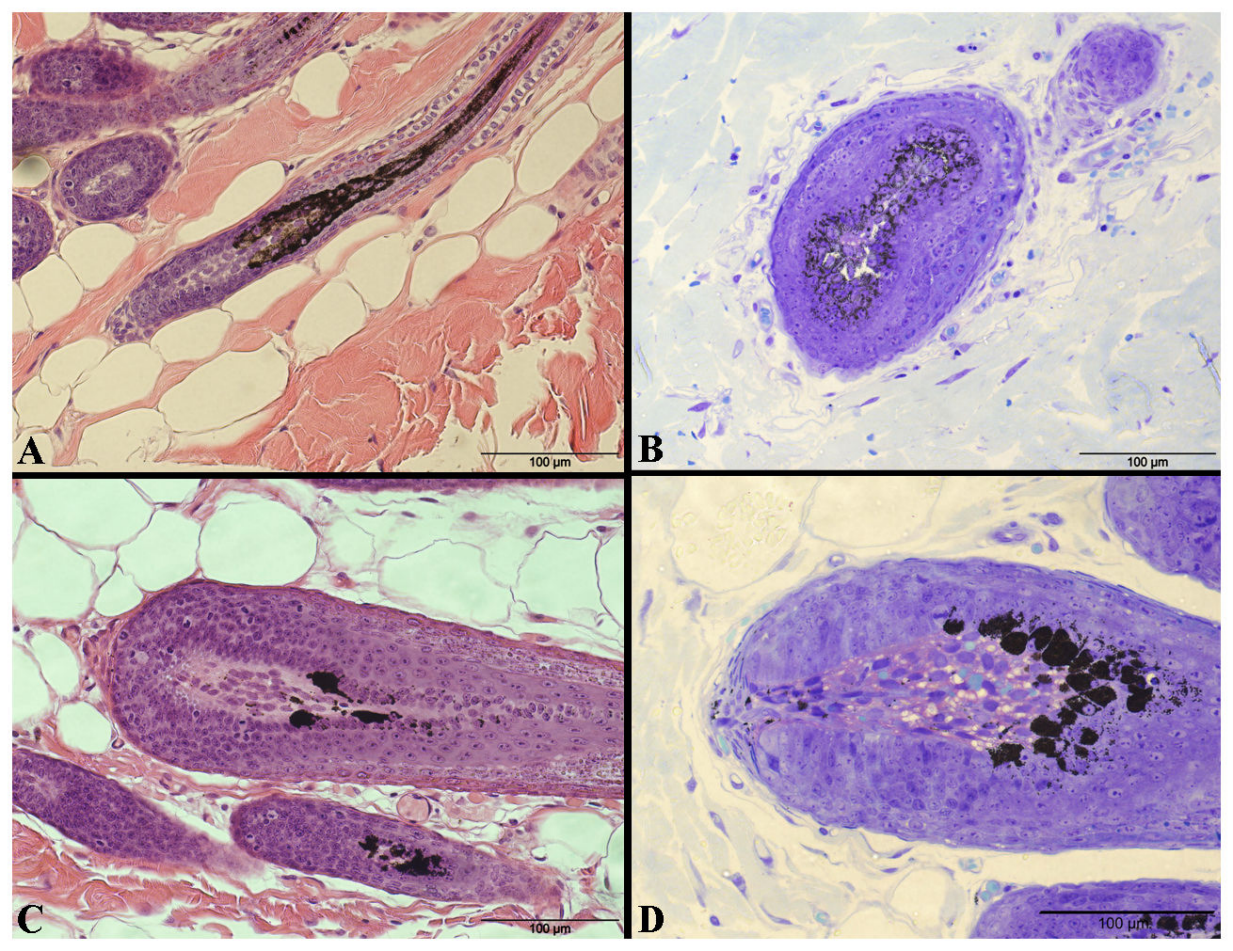
Representative light microscopic comparison of black (A, B) and blue (C, D) rabbit skin samples. HE-staining (A, C) and Toluidine blue O staining (B, D) was performed. Sagittal sections through hair bulbs are shown in A, C, D, and horizontal section through hair bulb is shown in B. Note the uneven distribution pattern of melanin granules and melanin clumps in hair matrix cells of hair bulbs of blue rabbit skin samples. Scale bars = 100 µm.

**Figure 3:**
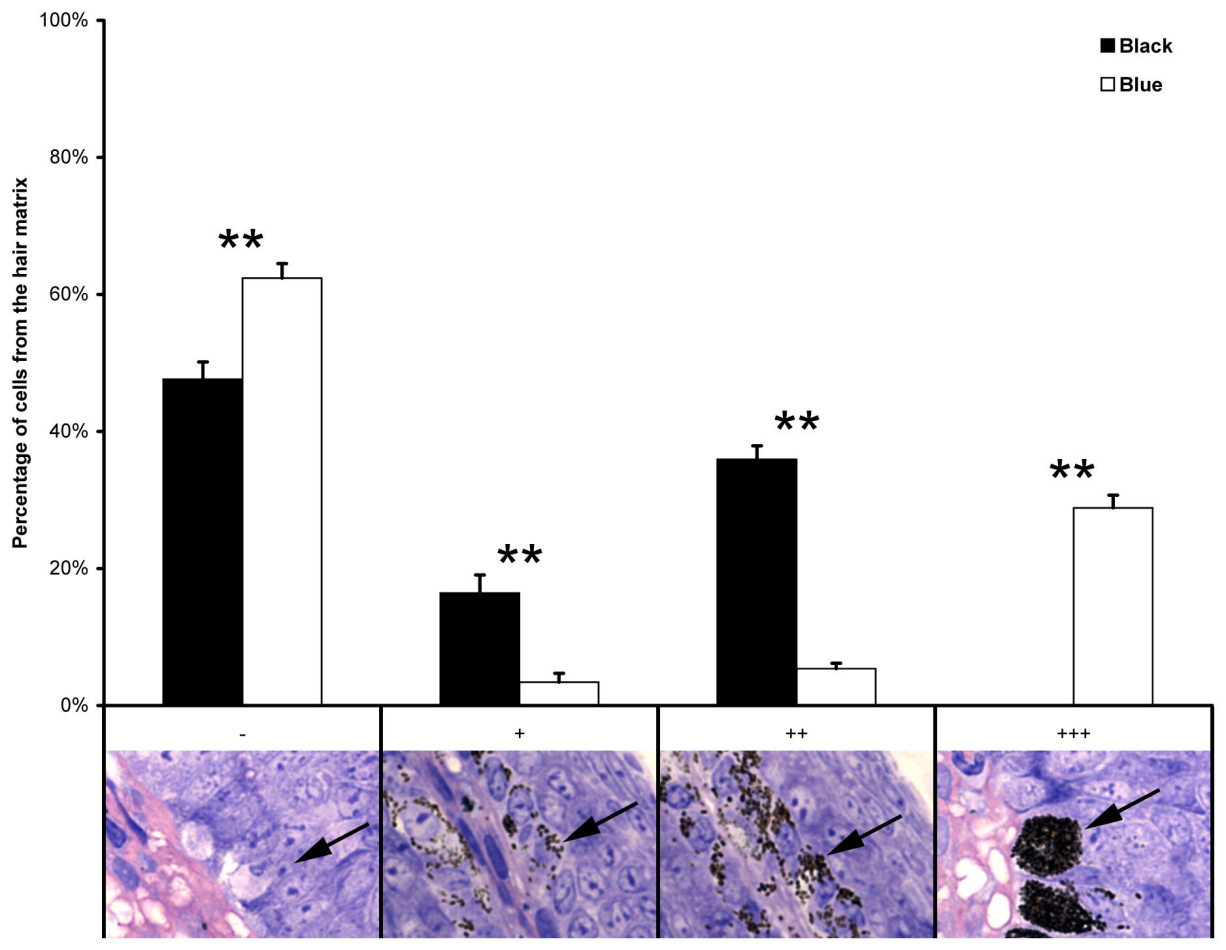
Proportions of cells with melanin granules in the hair matrix layer of hair bulbs for black and blue rabbits using a semi-quantitative analysis. The scoring system is: “-” = no melanin granules, “+” = few melanin granules, “++” = several melanin granules, “+++” = many melanin granules (several appear as melanin clumps). Scoring is represented in semi-thin sections (Technovit®) below the graph. Arrows point to characteristic cells of each category/score. Original magnification of insets is 63x. Statistical analysis revealed each single scores as significantly different (**, P-value<0.01) between black and blue rabbits.

Subsequently, histological analyses were performed using skin samples of black rabbits (n=6) and blue rabbits (n=4). Cells of the hair bulb matrix from black rabbits contained large numbers of fine melanin granules which are moderately and uniformly distributed in the cytoplasm. Nuclei in the horizontally sectioned hair bulb of black rabbits were free of melanin (Figure 2). The melanin granules in the hair bulbs from blue rabbits differ in several aspects from those in black ones. The majority of the melanin granules seem somewhat larger and form aggregates (melanin clumps). The pattern of melanin granules in hair matrix cells shows significant differences (P-value<0.01) between black and blue rabbit hair bulbs (Figure 3). These results are based on a qualitative scoring system, which was then semi-quantitatively analysed. It is important to note that blue rabbits only form these melanin clumps, yet in the blue rabbit hair bulbs, >90% of the hair matrix cells contain melanin clumps but no melanin granules. In contrast, approximately 50% of hair matrix cells of black rabbits contain either few or several melanin granules. Furthermore, the pattern of melanin granule distribution differs from dilute individuals as granule containing cells are uniformly distributed throughout the hair bulb matrix in black rabbits (Figures 2 and 3).

### Sequencing MLPH and Mutation Analysis

**Figure 4:**
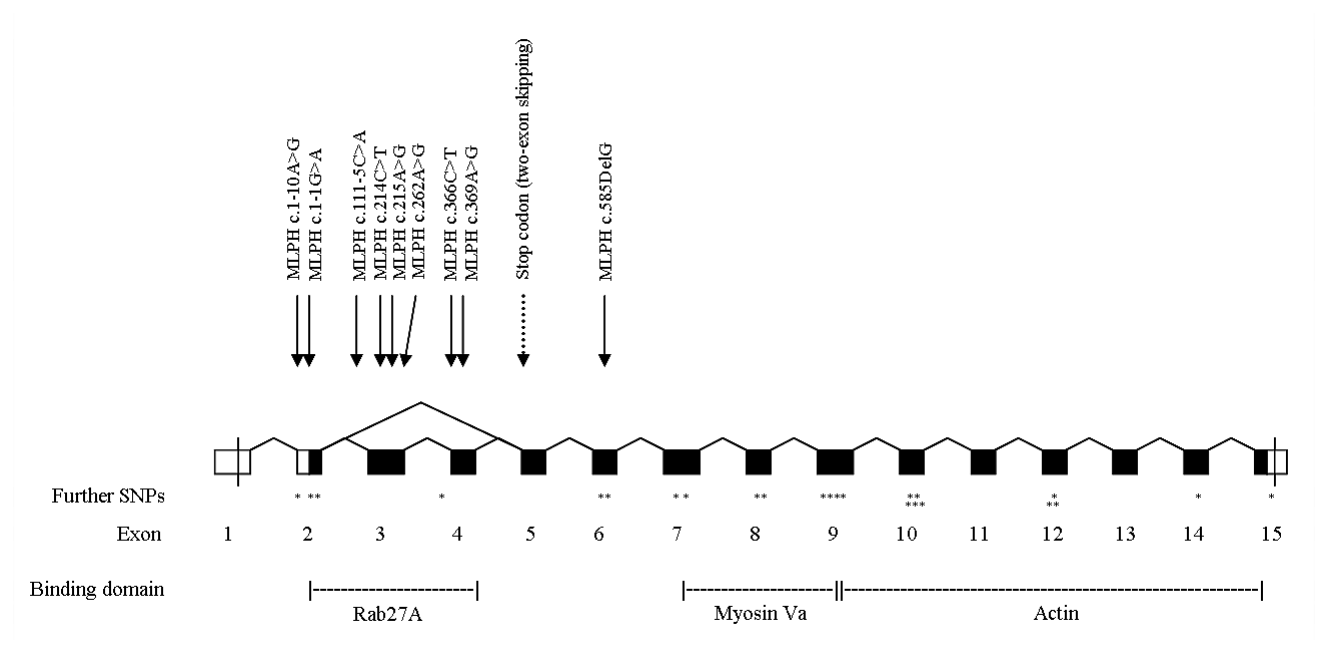
Structure of the melanophilin (MLPH) gene. Coding regions of exons are painted black, the 5’ and 3’ untranslated regions are white. The positions of the nine SNPs chosen for further analyses are indicated by arrows. Further SNPs detected within MLPH are indicated by asterisks. The premature stop codon caused by the skipping of exons 3 and 4 in dilute rabbits is indicated by a dashed arrow. The functional protein binding domains Rab27A, Myosin Va and Actin are given.

**Figure 5:**
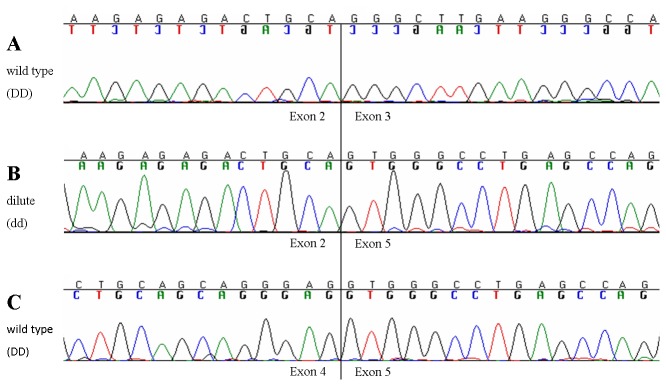
Comparisons of the cDNA sequences for melanophilin among a colour dilute rabbit and a black wild type rabbit. The sequences show the boundary of exon 2 to exon 3 (A) as well as of exon 4 to exon 5 (C) in the wild type rabbit in comparison with the sequence of a dilute rabbit (B), where exon 2 is spliced to exon 5. The vertical line marks the boundaries.

The *oryctolagus cuniculus* (OryCun2.0) map viewer of NCBI (http://www.ncbi.nlm.nih.gov/projects/mapview) locates rabbit *MLPH* on an unknown chromosome. *MLPH* is designated *LOC100343360*, which contains 35,141 bp genomic sequence. We ascertained the complete coding sequence of *MLPH* in one black (DD) and two dilute (dd) rabbits, including all exon-exon boundaries and 51 bp of the 5’ untranslated region (UTR) and 60 bp of the 3’ UTR (Figure 4). We identified a 1,689 bp open reading frame (ORF) in the cDNA of a black rabbit, which was translated into a protein of 563 amino acids. A total of 15 exons were detected in the DD-rabbits. In the cDNA of both dd-rabbits, however, exons 3 and 4 were not present and exon 2 was directly spliced to exon 5 (Figure 5). This exon skipping caused a frameshift resulting in a change of two amino acids (p.QGL[37-39]QWA) followed by a premature stop codon (p.K40*) (Figure S1). In the next step, we sequenced 570 bp genomic DNA containing 51 bp of intron 2, exon 3 (222 bp), intron 3 (92 bp), exon 4 (113 bp), and 92 bp of intron 4 in two black and two dilute rabbits. In total, 32 polymorphisms were detected within the exons of *MLPH* and two polymorphisms were detected within intron 2 and 3, respectively (Table 1). Of all exonic polymorphisms, twelve caused amino acid exchanges and were analysed for a potentially damaging effect. Two polymorphisms within exon 3 were classified as probably damaging (c.214CT, c.262AG). A deletion of one base pair within exon 6 (c.585delG), may be regarded as probably damaging as it causes a frameshift leading to a premature stop codon. This stop codon is located 166 triplets downstream of c.585delG in both dilute rabbits sequenced. In the case of the presence of the wild type allele at c.953TC, the stop codon is 123 triplets downstream of the deletion (Figure S1). Analysing the genomic DNA containing exon 3 and 4, no polymorphisms affecting a branch site or an invariant base of a splice site were found. However, we detected a SNP (c.111-5C>A) five base pairs upstream of the 5’end of exon 3 within the polypyrimidine tract. The pyrimidine base seems to be conserved at this position after comparing 35 species (Table S1). Only in chicken, a guanine is found at this location in some sequences. Another polymorphism located within the 5’ UTR of *MLPH* (c.1-10A>G) also seems to be located at a position conserved in mammals and marsupials (Table S1).

**Table 1:** SNPs detected within melanophilin (*MLPH*), their location, predicted effects on the protein sequence and protein function are given.

Polymorphism	Location	Amino acid	Predicted effect
	within	exchange	
	*MLPH*		
c.1-11C>T	exon 2	-	
c.1-10A>G	exon 2	-	
c.1-1G>A	exon 2	-	
c.48AG	exon 2	-	
c.52GA	exon 2	p.V18I	Benign
c.111-5C>A	intron 2	(p.Q37QfsX4)	Truncated protein
c.214CT	exon 3	p.Q72W	Probably damaging
c.215AG	exon 3	p.Q72R	Benign
c.262AG	exon 3	p.T88A	Probably damaging
c.333-17insGC	intron 3	-	
c.366CT	exon 4	-	
c.369AG	exon 4	-	
c.585delG	exon 6	p.L195LfsX123*	Truncated protein
		p.L195LfsX166*	
c.610TC	exon 6	p.R204W	Benign
c.642CG	exon 6	-	
c.718AG	exon 7	p.T240A	Benign
c.774AT	exon 7	-	
c.941GC	exon 8	p.G314A	Possibly damaging
c.953TC	exon 8	p.V318A	Benign
c.1029AG	exon 9	-	
c.1041GA	exon 9	-	
c.1092CT	exon 9	-	
c.1143TC	exon 9	-	
c.1197TC	exon 10	-	
c.1198AG	exon 10	p.G400S	Benign
c.1206AG	exon 10	-	
c.1209TC	exon 10	-	
c.1284AG	exon 10	-	
c.1440CG	exon 12	-	
c.1462GA	exon 12	p.A488D	Possibly damaging
c.1463AG	exon 12	p.A488D	Possibly damaging
c.1482AG	exon 12	-	
c.1602CT	exon 14	-	
c.1689+45GC	exon 15	-	

Predicted effects on protein function were obtained using PolyPhen2 software. The c.111-5C>A mutation presumably causes dilution in rabbits due to two-exon skipping.

^*^ Depending on the c.953TC genotype

To exclude the presence of an inversion, which might include exon 3 and 4 and therefore cause the two-exon skipping, we performed a long range PCR with the forward primer located within exon 3 and the reverse primer located within exon 5. In the two black as well as in the two dilute rabbits, we obtained PCR products of the appropriate size. Therefore, an inversion affecting this region could be excluded. 

All polymorphisms with predicted probably damaging effects (c.214CT, c.262AG, c.585delG) as well as the two polymorphisms located within conserved, non-coding regions (c.1-10A>G, c.111-5C>A) and four further polymorphisms located within exon 2 (c.1-1G>A), exon 3 (c.215AG) or exon 4 (c.366CT, c.369AG) were analysed in all 23 rabbits of the breeding trial. The SNPs c.1-1G>A, c.111-5C>A, c.366CT, c.369AG, and c.585delG showed associations with the dilute phenotype and were subsequently genotyped in further 30 rabbits of several breeds, which were not related with those of the breeding trial. Of these rabbits, seven showed the dilution phenotype, two rabbits showed a coat color which was classified as dark-dilute and 21 rabbits were wild type colored. The polymorphisms c.1-1G>A, c.111-5C>A, c.366CT, and c.369AG were highly correlated with each other at r^2^>0.9 (Figure S2). 

### Association Analysis

We performed a case-control analysis for the SNPs c.1-1G>A, c.111-5C>A, c.366CT, c.369AG, and c.585delG with coat colour dilution (Table S2). The P-values for association with the dilution phenotype were lowest for c.111-5C>A (1.5x10^-10^ to 6.8x10^-7^) and c.585delG (1.3x10^-10^ to 1.8x10^-7^). The c.111-5C>A mutation was homozygous in 14/15 colour diluted rabbits and the c.585delG deletion in 13/15 colour diluted rabbits (Table 2). Out of the 15 colour diluted rabbits, twelve individuals were homozygous for the mutations, c.111-5C>A and c.585delG. One colour diluted individual homozygous for the A/A genotype at c.111-5C>A, carried the wild type alleles (wt/wt) at c.585delG. A further at c.111-5C>A homozygous A/A rabbit was heterozygous at c.585delG. This rabbit showed a darker blue coat color and was classified as dark-dilute. The same dark-dilute phenotype was seen in a rabbit being heterozygous A/C at c.111-5C>A, but homozygous del/del at c.585delG. The c.953TC mutation was not conclusive for distinguishing dilute and dark-dilute phenotypes (Table S3). 

**Table 2:** Distribution of the melanophilin (*MLPH*) c.111-5C>A and c.585delG mutations in rabbits of different breeds.

Rabbit breed	Fur color	Dilute genotype	Number of animals	SNP c.111-5C>A			SNP c.585delG		
				C/C	A/C	A/A	w/w	w/del	del/del
Netherland	Wild Type	D-	11		x			x	
Dwarf	Wild Type	D-	2		x		x		
	Wild Type	D-	2	x			x		
	Dilute	dd	5			x			x
Lionhead	Wild Type	D-	1		x			x	
Dwarf	Wild Type	D-	2		x		x		
Loh	Wild Type	D-	2	x			x		
Netherland	Wild Type	D-	5		x			x	
Dwarf x Loh	Wild type	Dd	5		x			x	
	Dilute	dd	4			x			x
Vienna Blue	Dilute	dd	2			x			x
Dwarf Lop	Wild Type	D-	2	x			x		
	Wild Type	D-	2		x			x	
	Dilute	dd	1			x			x
Giant Lop	Wild Type	D-	1	x			x		
Checkered	Wild Type	D-	1		x		x		
Giant									
Rex	Wild Type	D-	1		x			x	
Angora	**Dilute**	**dd**	**1**			**x**	**x**		
Lionhead	**Dark-dilute**	dd*	**1**		**x**				**x**
Dwarf									
Loh	**Dark-dilute**	dd*	**1**			**x**		**x**	

The dilution status and genotype with corresponding number of animals and SNP genotypes are given. Bold faced letters indicate the dilute individuals, in which SNP genotypes differ between c.111-5C>A and c.585delG. The two individuals with less obvious dilution status are given at the bottom of the table. Their dilution status is indicated as “dark-dilute” (dd*).

The alleles of the SNPs c.366CT and c.369AG were in high linkage disequilibrium with the alleles of c.111-5C>A, but not as strongly associated with the colour dilution as the c.111-5C>A mutation. 

Therefore, genotyping the c.111-5C>A and c.585delG mutations in parallel allowed detection of the dilute phenotype in all rabbits analysed in this study. The c.111-5C>A dilution allele was homozygously or heterozygously detected in the breeds Netherland Dwarf, Lionhead Dwarf, Vienna Blue, Dwarf Lop, Checkered Giant, Rex, Angora, and Loh.

## Discussion

In the present study we detected mutations within *MLPH*, co-segregating with the dilution phenotype in the nine rabbit breeds tested. *MLPH* was selected as candidate gene due to histological as well as clinical examination of the rabbits within a breeding group of Netherland Dwarf, Loh, and Lionhead Dwarf rabbits and their crosses. Only in blue rabbits, our histological analysis revealed clumps of melanin in hair matrix cells as well as a non-uniformly distribution pattern of the melanin granules throughout the hair bulb matrix. No abnormal clinical findings could be ascertained in the rabbits examined in the present study. Pigmentary dilution due to large agglutinations of pigment in the hair shafts and accumulation of melanosomes in melanocytes are characteristics of human Griscelli syndromes [11]. The dilution phenotype of the human Griscelli syndrome type 3 caused by *MLPH* mutations is usually not accompanied by severe clinical diseases [21]. This is in consistency with observations in the mouse [23]. Therefore, *MLPH* was sequenced as it was the most likely functional candidate gene. 

The polymorphism c.111-5C>A is the most probable cause for dilute coat color in rabbits. Skipping of two exons was detected in 14/15 dilute rabbits, which is likely caused by the polymorphism c.111-5C>A. The exon skipping causes a premature stop codon at the fourtieth amino acid of the transcript (p.Q37QfsX4), while c.585delG is located further downstream. Both mutations are in most, but not all cases within the same linkage phase. In a single case of a dark-dilute rabbit showing a heterozygous genotype at c.111-5C>A and the homozygous deletion at c.585delG, the latter mutation might be of relevance, as it also leads to a frameshift and an altered amino acid sequence with a premature stop codon 123 or 166 amino acids downstream (p.L195LfsX123 or p.L195LfsX166), respectively (dependent on the polymorphism c.953TC). The c.585delG polymorphism therefore might have an effect on coat colour dilution when the individual has not the homozygous A/A genotype at c.111-5C>A. 

Though c.366CT and c.369AG were in high linkage disequilibrium with c.111-5C>A, their wild type allele is not conserved among different species (Table S1). Therefore, they are not supposed to influence the exon skipping through, for example, the destruction of an exonic splicing enhancer. Further variants classified as potentially damaging and located within conserved regions of *MLPH* showed only low associations with the dilution phenotype. They may be assumed to have arisen subsequently to the causal mutations, due to missing selection pressure. 

The *MLPH* two-exon skipping seen in dilute rabbits leads to a frameshift and results in a change in the amino acid sequence (p.QGL[37-39]QWA) followed by a premature stop codon (p.K40*). This mutation eliminates the myosin Va and the actin binding domain and leaves only 37/153 aa of the region containing the RAB27A binding domain [14,25,26]. As for the coordination of melanosome transport, and distribution, a tripartite protein complex of Rab27A, melanophilin, and Myosin Va is essential [14], and it can be assumed that the p.Q37QfsX4 protein is not functional. In case of a normal transcript without exon skipping, the frameshift mutation c.585delG (p.L195LfsX123 or p.L195LfsX166) also causes a change of the amino acid sequence and truncation of the protein, affecting the complete myosin Va and actin binding domain [25].

Mutations for the diluting genotype were determined in several other species. In cats, dilution of the coat color is caused by the deletion of one base-pair within exon 2, leading to a premature stop codon eleven amino acids downstream [26]. This mutation therefore shows a similarly truncated protein like the one in the present study. Most of the further mutations causing a dilution phenotype in different species are single base exchanges within the RAB27A binding domain. In lavender chicken and also in human Griscelli syndrome type 3, the causal mutation is a R35W substitution [3,22]. Leaden colored mice show a deletion of 6 amino acids on positions 31 to 37 [23]. In dogs and quails, however, other mutations have been described. In dogs, a polymorphism at the last nucleotide of the first, untranslated exon of *MLPH* was completely associated with coat color dilution [27] and in quails, the causal mutation is quite complex, as it includes different chromosomal rearrangements, which affect *MLPH* as well as three further genes [28]. 

The c.111-5C>A mutation appears as the most probable candidate to cause the two-exon skipping in dilute rabbits. It is located within the splice acceptor polypyrimidine tract of *MLPH* intron 2, five base pairs upstream of the start of exon 3, at a location which is supposed to contain a pyrimidine base. The percentage of occurrence of a pyrimidine in this position is at 87% in vertebrate genes [29]. While exon skipping due to mutations of the splice acceptor site usually affect the two invariant nucleotides A and G at positions -1 and -2, exon skipping defects resulting from mutations at positions -14 to -3 (Y_(10_)NC, [29]) are rare. However, mutations affecting different positions within the polypyrimidine tract have been previously described to cause exon skipping. A T>A mutation at position -6 within *GHR* was recently described to cause exon skipping by disruption of the polypyrimidine tract [30]. Further mutations causing partial or complete skipping of the subsequent exon were reported for the positions -11 (T>A [31]) within *MLH1*, and two mutations within intron 2 of *ß globin* at -8 (T>G [32-33] and -7 (C>G [34]), respectively. The polypyrimidine tract has been shown to be of importance during the splicing process and for branch site definition [35,36]. Efficient splicing depends on the AG dinucleotide at the 3' splice junction, but also on the length of the polypyrimidine stretch [37]. The total length of the polypyrimidine tract seems to play a major role for the impact of a pyrimidine to purine exchange within this tract [38]. Furthermore, the thymine content of the polypyrimidine tract may affect splicing efficiency [39]. The polypyrimidine tract of the second intron of *MLPH* counts 19 pyrimidines in rabbits, which is of medium size [37], including seven thymines and twelve cytosines. This number of thymines in the polypyrimidine tract seems low, as the probability of a thymine is higher than that of cytosine for the complete polypyrimidine tract except for position -6 [29]. Therefore, the polypyrimidine tract might already be weak. In dilute rabbits, the last cytosine at position -5 is changed to adenine, which might impede the splicing process. However, the effects of mutations affecting the polypyrimidine tract are not completely understood and also in silico predictions of mutation effects in this region have to be experimentally confirmed [30].

A further characteristic in the present study is the skipping of not only the third, but also of the fourth exon. Missing of more than one exon can be due to an inversion or a deletion of genomic DNA [40]. This was excluded in the present study by sequencing of genomic DNA and long range PCR. Two-exon skipping can also occur because of an alternatively spliced adjacent exon [41], which is also not known for exon 3 and 4 of *MLPH*. The remaining cases previously described are rare and due to splice mutations [40,42-46]. A two-exon skipping was explained by the order of intron removal [44]. A splice acceptor mutation caused skipping of exons 5 and 6 of *COL5A1* due to rapid removal of intron 5 from the transcript relative to introns 4 and 6, which left a large composite exon 5/exon 6 construct that was skipped entirely. This might be also the case in the present study, as intron 3 between the two skipped exons only comprises 92 base pairs and therefore is the smallest within the complete gene, while intron 2 and 4 contained more than 6000 and 4500 base pairs, respectively. It may be assumed, that exons 3 and 4 fuse early during the splicing process and subsequently the c.111-5C>A mutation prevents splicing of the combined exon structure. Another explanation of a two-exon skipping was based on the detection of a splice donor (+5) mutation within intron 13 of *OXCT1*. This splice donor mutation led to retention of intron 13, thus causing the retention of intron 11 and 12. This so called splicing paralysis resulted in skipping the introns 11-13 and exons 12 and 13 [46]. In our data, we could not see retention of an intron in cDNA and thus, this latter explanation seems unlikely for the *MLPH*-associated dilute phenotype in rabbits. 

In conclusion, the dilution phenotype in rabbits is likely caused by a two-exon skipping of exon 3 and 4 and a frameshift of the open reading frame causing a premature stop codon (p.Q37QfsX4). The c.111-5C>A mutation, located within intron 2 of *MLPH*, is the most probable cause for this exon skipping and therefore for the dilute phenotype in rabbits. The dilute phenotype seems to be further influenced by another frame-shift mutation (c.585delG), but only one individual was exclusively homozygous for this genotype. This deletion does not affect the RAB27A binding domain like the c.111-5C>A mutation. The mutations detected herein in rabbits expand the contingent of known dilution mutations in different species and provide an additional view on the splicing mechanism.

## Materials and Methods

### Ethics Statement

 All animal work has been conducted according to the national and international guidelines for animal welfare. The Lower Saxony state veterinary office at the Niedersächsisches Landesamt für Verbraucherschutz und Lebensmittelsicherheit, Oldenburg, Germany, was the responsible Institutional Animal Care and Use Committee (IACUC) for this specific study. The breeding experiment and EDTA-blood sampling of the rabbits for the present study had been approved by the IACUC of Lower Saxony, the state veterinary office Niedersächsisches Landesamt für Verbraucherschutz und Lebensmittelsicherheit, Oldenburg, Germany (registration number 33.9-42502-04-11/0563). Tissue samples were taken from rabbits, which had to be euthanized due to other medical reasons (after inducing a deep anesthesia by intramuscular injection of 5 mg/kg xylazine and 35 mg/kg ketamine, 2 ml of T61 was applied intrapulmonal for euthanasia). For blood or hair sampling of living animals, no anesthesia was applied as this would have meant at least equivalent stress than taking a sample. Each rabbit was only sampled once. Samples from rabbits of the further breeds were taken in the context of diagnostic analyses by a veterinarian at the Clinic for Pets, Reptiles and Pet and Feral Birds, University of Veterinary Medicine Hannover. We obtained informed consent from all owners of the rabbits. 

### Animals

We obtained blood or hair root samples of a total of 53 rabbits, whereof 23 animals were rabbits of the breeding trial (Figure S3). In this breeding trial, a Netherland Dwarf male showing fur dilution (blue coat color) and descending from a dilute x non-dilute mating was crossed with two black Loh females and one black Lionhead Dwarf female. None of the descendants showed diluted fur. Two Loh x Netherland Dwarf females were again crossed with the dilute Netherland Dwarf male. Of the resulting offspring, four individuals showed blue fur, five had black fur and one was completely white, which rendered a phenotypical determination of the dilution phenotype impossible. In total, of all these 23 rabbits, six showed fur dilution. 

All rabbits were examined by veterinarians at the Clinic for Pets, Reptiles and Pet and Feral Birds and at the Institute for Animal Breeding and Genetics, University of Veterinary Medicine Hannover Foundation for defects accompanying the dilute coat color like in dogs [8,9,23] or in human Griscelli syndrome of types 1 [17] and 2 [18]. No difference in the health status was observed between rabbits with dilute and non-dilute color. The rabbits were between six months and four years of age when examined, the oldest dilute rabbit was three and a half years old. 

The other 30 rabbits were from several breeds (Netherland Dwarf, Loh, Lionhead Dwarf, Vienna Blue, Rex, Angora, Dwarf Lop, Giant Lop, Checkered Giant). Of these rabbits, 21 were wild type colored, seven showed dilute fur color and two rabbits showed a darker blue or blue-brownish coat color, which was classified as dark-dilute and might be a variant of the dilution phenotype. 

Genomic DNA was extracted from EDTA-blood or hair samples using routine procedures. Tissue samples for the RNA extraction were skin tissue of one black and two dilute rabbits. These samples were stored in RNAlater RNA Stabilization Reagent (Qiagen, Hilden, Germany) for stabilization and protection of cellular RNA *in situ* for storing at -20°C. The RNA was extracted from tissue samples using the RNeasy Lipid Tissue Mini Kit (Qiagen) according to manufacturer's protocol and transcribed into cDNA using the Maxima First Strand cDNA Synthesis Kit for RT-qPCR (Fermentas Life Sciences, St. Leon-Rot, Germany).

### Histological analysis

Skin samples of six black and four blue rabbits were fixed in Bouin’s solution and Ca-formol and were embedded in paraffin wax (Paraplast Standard, Leica Microsystems, Wetzlar, Germany) according to standard protocols [47]. The paraffin blocks were sectioned with a rotary microtome (Leica Microsystems, Wetzlar, Germany), and sections not thicker than 5 μm were used for staining.

Moreover, small Ca-formol fixed tissue blocks of about 0.5 mm^3^ were prepared, carefully dehydrated with graded ethanol (70, 80, 90, 2×96, 2×100%) and embedded in the water-soluble and rather shrinkage-free 2-hydroxy-methacrylate Technovit 7100 (Heraeus-Kulzer, Wehrheim, Germany [48,49]). Two µm plastic sections were cut with a motor-driven rotation microtome (Model 1140, Autocut, Reichert-Jung) and transferred to slides. 

Paraffin sections of all collected samples were stained with haematoxylin and eosin (H&E, haematoxylin according to Delafield) and plastic sections were stained with Toluidine blue O [47].

All skin samples (six black rabbits, four blue rabbits) were investigated semi-quantitatively for their melanin granule distribution in hair matrix cells. A total of sixty longitudinal histological hair bulb sections per phenotype were separately evaluated by three scientists using a qualitative scoring system. The scoring system distinguished four categories including “-” = no melanin granules, “+” = few melanin granules, “++” = several melanin granules, “+++” = many melanin granules (several appear as melanin clumps). Within each category, the presence of the respective score was then subjected to a statistical analysis using a one-way ANOVA test. P-values<0.05 were defined as significant with * = P-value<0.05 and ** = P-value<0.01.

### Sequence and mutation analysis

For mutation analysis of rabbit *MLPH*, we used cDNA of one black Loh and two dilute rabbits whereof one was a Netherland Dwarf and the other one was a cross of a Netherland Dwarf (75%) x Loh (25%). Four pairs of primers were designed based using the rabbit *MLPH* cDNA sequence obtained by Ensembl (www.ensembl.org) and the Primer3 software (http://frodo.wi.mit.edu/). These primers yielded products covering the complete *MLPH* open reading frame and all exon-exon boundaries, as well as 51 bp of the 5’ UTR and 60 bp of the 3’ UTR. The exons 3 and 4 of *MLPH*, which were skipped in dilute rabbit cDNA, were sequenced on genomic DNA of the same three animals used for cDNA sequence analysis and in addition, a black Netherland Dwarf x Loh crossbred with the genotype Dd was sequenced. For this purpose, two pairs of primers were designed as described above. To exclude an inversion including exon 3 and 4, we further performed a long range PCR on genomic DNA of two black (DD and Dd) and two dilute (dd) rabbits, with the forward primer located within exon 3 and the reverse primer located within exon 5. Products of the appropriate size in all individuals in combination with the genomic sequences containing exons 3 and 4 would exclude the presence of an inversion. All primer pairs are shown in Table S4. 

PCRs were carried out according to the standard protocol advised by the manufacturer of the *Taq*DNA polymerase (Qbiogene Heidelberg, Germany). Sequencing of the PCR products was performed using the ABI BigDye Terminator v3.1 sequencing kit (Life Technologies, Darmstadt, Germany). The products were analysed on an automated ABI 3500 capillary sequencer (Life Technologies). Mutation analysis was performed using Sequencher 4.8 (GeneCodes, Ann Arbor, MI, USA). The open reading frame was determined using Open Reading Frame Finder (http://www.ncbi.nlm.nih.gov/gorf/). Translation into protein sequence was done using CLUSTALW (http://www.genome.jp/tools/clustalw/). In the next step, all detected polymorphisms were analysed for their potentially damaging effect using PolyPhen2 [50]. All polymorphisms, which were classified as probably damaging and all polymorphisms located outside of the reading frame but within highly conserved areas were genotyped in the 23 rabbits from the breeding trial using PCR-RFLPs. Then all polymorphisms associated with the dilute phenotype were genotyped in all 52 rabbit samples with a colored coat. All primer pairs for genotyping are given in Table S4. Mismatch PCR was partly used to create restriction sites for specific enzymes [51]. Digestion took place using specific restriction enzymes (Table S4) and the resulting products were separated on agarose gels. Association of SNPs with the dilute phenotype was tested using the CASECONTROL procedure of SAS/Genetics (Statistical Analysis System, version 9.3, Cary, NC, USA, 2013). Statistical calculation of pairwise linkage disequilibrium (LD) was performed and pictured using HAPLOVIEW 4.0 [52].

### Accession Numbers

Sequence data has been deposited at the GenBank Data Libraries (KC791692, KC791692). 

## Supporting Information

Figure S1 **Comparison of the different variants of truncated melanophilin proteins in dilute rabbits (variants 2-4) with the complete protein in wild type rabbits (1).** Variant 2 is a result from skipping of exon 3 and 4 of MLPH (p.Q37QfsX4). Exon skipping is presumably caused by the c.111-5CγA transversion. Variants 3 and 4 (p.L195LfsX123 and p.L195LfsX166, respectively) are caused by the c.585delG mutation. Which one of these variants is generated depends on a further polymorphism affecting the premature stop codon of variant 3 (c.953T>C). The RAB27A domain is highlighted in green.
(DOC)

Figure S2 **Linkage disequilibrium (LD) of the nine SNPs selected for genotyping in the 23 rabbits of the breeding trial (c.1-10AγG, c.214A>G, c.215A>G and c.262A>G) and in all 53 rabbits of different breeds (c.1-1GγA, c.111-5CγA, c.366C>T, c.369A>G and c.585delG).** The latter five SNPs showed strong LD with the dilute phenotype. The pairwise r^2^-values are shown for each SNP pair. Red squares indicate complete linkage disequilibrium.
(DOC)

Figure S3 **Pedigree of the rabbits from the breeding trial and the haplotypes of nine polymorphisms genotyped for these animals.** Haplotypes include the polymorphisms c.1-10AγG, c.1-1GγA, c.111-5CγA, c.214A>G, c.215A>G, c.262A>G, c.366C>T, c.369A>G and c.585delG.
(DOC)

Table S1 **Conservation of nucleotides in different species at the melanophilin (*MLPH*) SNP positions detected in rabbits.** Nucleotides in brackets were located at position c.-9, but in that species, this position matched with c.1-10 in other species due to the surrounding sequence.
(DOC)

Table S2 **Case-control analysis for five melanophilin-associated polymorphisms with the dilution phenotype in 15 dilute and 37 colored non-dilute rabbits.** The χ^2^- and P-values are shown for genotype, allele and trend tests.
(DOC)

Table S3 **Distribution of the melanophilin (*MLPH*) c.111-5CγA, c.585delG and c.953T>C mutations in rabbits of different breeds.** The dilution status and genotype with corresponding number of animals and SNP genotypes are given. Bold faced letters indicate the dilute individuals, in which SNP genotypes differ between c.111-5C>A and c.585delG. The two individuals with less obvious dilution status are given at the bottom of the table. Their dilution status is indicated as “dark-dilute” (dd*).
(DOC)

Table S4 **Primer sequences with their product sizes (**P**) for amplification of sequences within melanophilin (*MLPH*) to be used for sequencing, long range PCR or genotyping as well as the targeted region and the appropriate template.** The primers were designed to work at an annealing temperature of 60°C. In the case of performing a mismatch PCR for digestion, the base pairs, which were changed within a primer relative to the reference sequence, are highlighted in gray. If the product of a pair of primers was enzymatically digested for genotyping, the enzyme used is given.
(DOC)
