# Supplementary material for: Two-Exon Skipping within MLPH Is Associated with Coat Color Dilution in Rabbits
Source: PLoS One. 2013 Dec 20;8(12):e84525. doi: 10.1371/journal.pone.0084525 (PMC3869861; doi:10.1371/journal.pone.0084525)
Supplement: Figure S1 — Comparison of the different variants of truncated melanophilin proteins in dilute rabbits (variants 2-4) with the complete protein in wild type rabbits (1). Variant 2 is a result from skipping of exon 3 and 4 of MLPH (p.Q37QfsX4). Exon skipping is presumably caused by the c.111-5CγA transversion. Variants 3 and 4 (p.L195LfsX123 and p.L195LfsX166, respectively) are caused by the c.585delG mutation. Which one of these variants is generated depends on a further polymorphism affecting the premature stop codon of variant 3 (c.953T>C). The RAB27A domain is highlighted in green. (DOC) [file pone.0084525.s001.doc]

1 MGRKLDLSKLTDDEARHVWEVVQRDFDLRRKEEERLQGLKGQIQRESSKRELLADTAHLNETH

2 MGRKLDLSKLTDDEARHVWEVVQRDFDLRRKEEERLQWA*****

3 MGRKLDLSKLTDDEARHVWEVVQRDFDLRRKEEERLQGLKGQIQRESSKRELLADTAHLNETH

4 MGRKLDLSKLTDDEARHVWEVVQRDFDLRRKEEERLQGLKGQIQRESSKRELLADTAHLNETH

1 CARCLRPYQLLVNSRRQCLHCGLFTCRSCSRAHPEEQGWLCDPCHLARVVKMGSLEWYYEHVR

2

3 CARCLRPYQLLVNSRRQCLHCGLFTCRSCSRAHPEEQGWLCDPCHLARVVKMGSLEWYYEHVR

4 CARCLRPYQLLVNSRRQCLHCGLFTCRSCSRAHPEEQGWLCDPCHLARVVKMGSLEWYYEHVR

1 ARFKRFGSAKVIRSLYGRLQQGGGPEPGLEERSGDSDLADEHGEPDAGAQAPPLGTKKKRLLS

2

3 ARFKRFGSAKVIRSLYGRLQQGGGPEPGLEERSGDSDLADEHGEPDAGAQAPPLGTKKKRLLS

4 ARFKRFGSAKVIRSLYGRLQQGGGPEPGLEERSGDSDLADEHGEPDAGAQAPPLGTKKKRLLS

1 IHDLDLEADSDHSTWSCGQLLDLSSEAEATGSLQSLTGEPHTWETTSQETTVLEEADPGASEC

2

3 IHDLDLRRTRITPRGPVVSSWTCPPRLRPRAACSPSQVSPTPGRPPPRRRPSWRKLTQGPPSA

4 IHDLDLRRTRITPRGPVVSSWTCPPRLRPRAACSPSQVSPTPGRPPPRRRPSWRKLTQGPPSA

1 QPPAEPPGGLSPSRREAPKEPCQPGTTSSTGLGLAAAPGTASGSSGQLPLQCQADVDTSDEGG

2

3 SPLQNHRAASRPPDGRPPRSPASPEPPAARAWGLPPLQGRPLAAVDSSPCSARPTWTPLTKEA

4 SPLQNHRAASRPPDGRPPRSPASPEPPAARAWGLPPLQGRPLAAVDSSPCSARPTWTPLTKEA

1 TGVASAAGPHPRRWSWTSSDSQPPAAGPHSAADTEEEALRKRLEELTSNVSDQEEEEGEPAGG

2

3 LG*

4 LGQPRRLAPTPDAGAGPRLTVSPQLPGHTQRRTRRRRPSGKGWRS*

1 DHAGALPPRSRQDPAQVCTDASPTPGRGQSPQGAGGPAQPSRSTDEQLSALEDRVAAAASEVQ

2

3

4

1 QAESEVSAIESRIAALRAAGLTVKPSGKPRRKSNLPIFVPRLLGTPDKRPEDPDTEPPGEAKD

2

3

4

1 ETVPYLLRRKYSPRGQGKDEVSLDRKSPYRGSLTQRNPSRRRGAASAIFTKPVMAQQP*****

2

3

4

**Figure S1. Comparison of the different variants of truncated melanophilin proteins in dilute rabbits (variants 2-4) with the complete protein in wild type rabbits (1).** Variant 2 is a result from skipping of exon 3 and 4 of *MLPH* (p.Q37QfsX4). Exon skipping is presumably caused by the c.111-5C>A transversion. Variants 3 and 4 (p.L195LfsX123 and p.L195LfsX166, respectively) are caused by the c.585delG mutation. Which one of these variants is generated depends on a further polymorphism affecting the premature stop codon of variant 3 (c.953T>C). The RAB27A domain is highlighted in green.
