# Supplementary material for: Two-Exon Skipping within MLPH Is Associated with Coat Color Dilution in Rabbits
Source: PLoS One. 2013 Dec 20;8(12):e84525. doi: 10.1371/journal.pone.0084525 (PMC3869861; doi:10.1371/journal.pone.0084525)
Supplement: Figure S2 — Linkage disequilibrium (LD) of the nine SNPs selected for genotyping in the 23 rabbits of the breeding trial (c.1-10AγG, c.214A>G, c.215A>G and c.262A>G) and in all 53 rabbits of different breeds (c.1-1GγA, c.111-5CγA, c.366C>T, c.369A>G and c.585delG). The latter five SNPs showed strong LD with the dilute phenotype. The pairwise r2-values are shown for each SNP pair. Red squares indicate complete linkage disequilibrium. (DOC) [file pone.0084525.s002.doc]

**
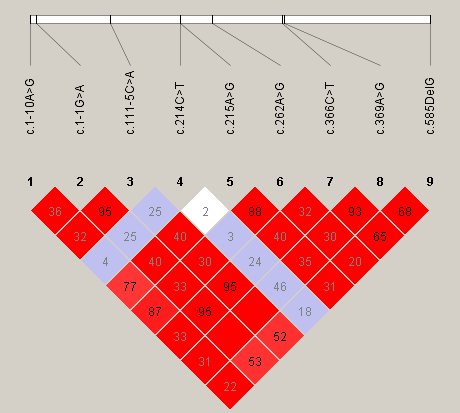
**

**Figure S2.** **Linkage disequilibrium (LD) of the nine SNPs selected for genotyping in the 23 rabbits of the breeding trial (c.1-10A>G, c.214A>G, c.215A>G and c.262A>G) and in all 53 rabbits of different breeds (c.1-1G>A, c.111-5C>A, c.366C>T, c.369A>G and c.585delG).** The latter five SNPs showed strong LD with the dilute phenotype. The pairwise r^2^-values are shown for each SNP pair. Red squares indicate complete linkage disequilibrium.
