# Supplementary material for: Two-Exon Skipping within MLPH Is Associated with Coat Color Dilution in Rabbits
Source: PLoS One. 2013 Dec 20;8(12):e84525. doi: 10.1371/journal.pone.0084525 (PMC3869861; doi:10.1371/journal.pone.0084525)
Supplement: Figure S3 — Pedigree of the rabbits from the breeding trial and the haplotypes of nine polymorphisms genotyped for these animals. Haplotypes include the polymorphisms c.1-10AγG, c.1-1GγA, c.111-5CγA, c.214A>G, c.215A>G, c.262A>G, c.366C>T, c.369A>G and c.585delG. (DOC) [file pone.0084525.s003.doc]

**
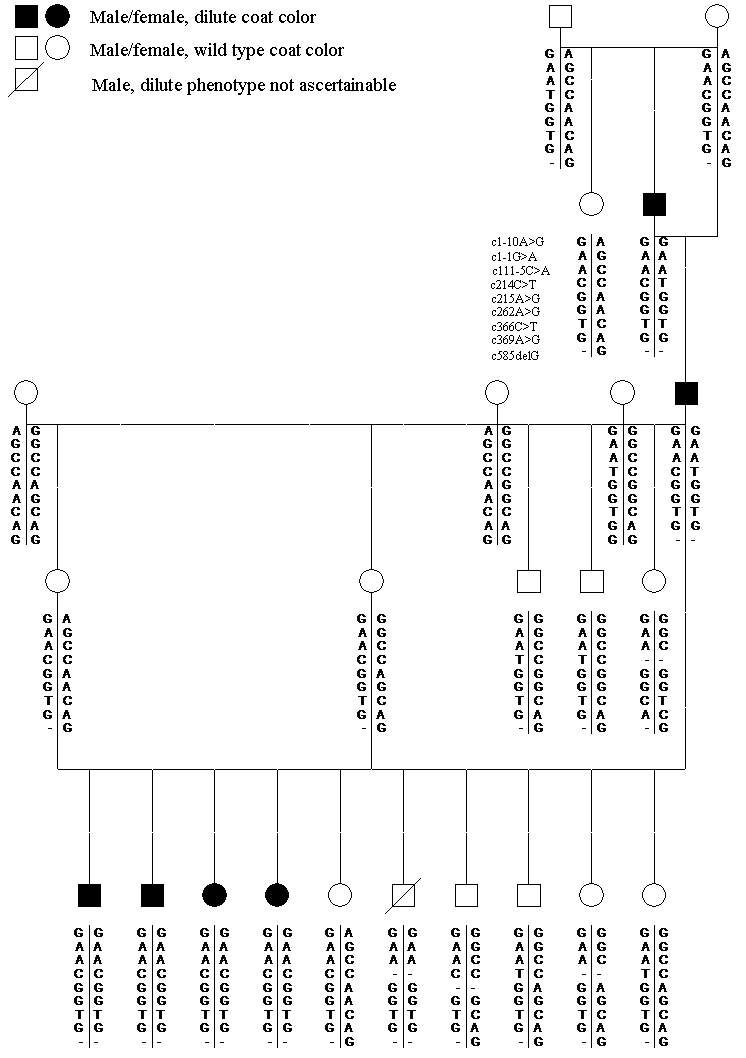
**

**Figure S3.** **Pedigree of the rabbits from the breeding trial and the haplotypes of nine polymorphisms genotyped for these animals.** Haplotypes include the polymorphisms c.1-10A>G, c.1-1G>A, c.111-5C>A, c.214A>G, c.215A>G, c.262A>G, c.366C>T, c.369A>G and c.585delG.
