# Supplementary material for: Two-Exon Skipping within MLPH Is Associated with Coat Color Dilution in Rabbits
Source: PLoS One. 2013 Dec 20;8(12):e84525. doi: 10.1371/journal.pone.0084525 (PMC3869861; doi:10.1371/journal.pone.0084525)
Supplement: Table S1 — Conservation of nucleotides in different species at the melanophilin (MLPH) SNP positions detected in rabbits. Nucleotides in brackets were located at position c.-9, but in that species, this position matched with c.1-10 in other species due to the surrounding sequence. (DOC) [file pone.0084525.s004.doc]

**Table S1.** **Conservation of nucleotides in different species at the melanophilin (*MLPH*) SNP positions detected in rabbits.** Nucleotides in brackets were located at position c.-9, but in that species, this position matched with c.1-10 in other species due to the surrounding sequence.

|  | c.1-10 | c.1-1 | c.111-5 | c.214 | c.215 | c.262 | c.366 | c.369 | c.585 |
| --- | --- | --- | --- | --- | --- | --- | --- | --- | --- |
| Rabbit (black) | A | G | C | C | A | A | C | A | Wt |
| Rabbit (gray) | G | A | A | T | G | G | T | G | DelG |
| Human | A | A | C | C | A | A | T | G | Wt |
| Gorilla | A | A | C | C | G | A | T | G | Wt |
| Gibbon | A | A | C | C | G | A | T | G | Wt |
| Tarsier | A | A | T | C | G | A | T | G | Wt |
| Mouse | A | G | C | C | G | G | C | G | Wt |
| Rat | A | G | C | C | G | G | C | G | Wt |
| Ferret | G (A) | G | C | C | G | A | C | G | Wt |
| Squirrel | A | G | T | C | G | A | C | A | Wt |
| Tree Shrew | A | G | C | C | G | G | C | G | Wt |
| Guinea Pig | A | G | C | C | G | A | T | G | Wt |
| Cat | A | G | C | C | G | A | C | G | Wt |
| Dog | A | G | C | C | G | A | C | G | Wt |
| Cow | A | G | T | C | G | A | C | C | Wt |
| Horse | A | G | C | C | G | A | C | G | Wt |
| Elephant | A | G | C | C | G | A | C | G | Wt |
| Panda | A | G | C | C | G | A | C | G | Wt |
| Microbat | A | G | C | C | G | A | C | G | Wt |
| Tasmanian Devil | A | A | C | C | G | A | T | A | Wt |
| Opossum | C (A) | A | C | C | G | A | C | G | Wt |
| Wallaby | A | A | C | C | G | A | T | G | Wt |
| Platypus | A | G | C | A | A | A | C | A | Wt |
| Chicken | C | G | G | A | A | A | C | G | Wt |
| Zebra Finch | G | G | T | A | A | A | C | A | Wt |
| Turkey | T | G | T | A | A | A | C | G | Wt |
| Anole Lizard | C | A | T | A | A | A | T | G | Wt |
| Chinese softshell turtle | T | G | T | A | A | A | T | G | Wt |
| Xenopus | T | C | T | C | A | G | T | T | Wt |
