# Supplementary material for: Two-Exon Skipping within MLPH Is Associated with Coat Color Dilution in Rabbits
Source: PLoS One. 2013 Dec 20;8(12):e84525. doi: 10.1371/journal.pone.0084525 (PMC3869861; doi:10.1371/journal.pone.0084525)
Supplement: Table S2 — Case-control analysis for five melanophilin-associated polymorphisms with the dilution phenotype in 15 dilute and 37 colored non-dilute rabbits. The χ2- and P-values are shown for genotype, allele and trend tests. (DOC) [file pone.0084525.s005.doc]

**Table S2. Case-control analysis for five melanophilin-associated polymorphisms with the dilution phenotype in 15 dilute and 37 colored non-dilute rabbits.** The χ^2^- and P-values are shown for genotype, allele and trend tests.

| SNP | χ^2^ | | | P-value | | |
| --- | --- | --- | --- | --- | --- | --- |
|  | Genotype | Allele | Trend | Genotype | Allele | Trend |
| c.1-1G>A | 31.98 | 20.97 | 24.60 | 1.14x10^-7^ | 4.66x10^-6^ | 7.06x10^-7^ |
| c.111-5C>A | 45.20 | 24.67 | 33.55 | 1.53x10^-10^ | 6.81x10^-7^ | 6.94x10^-9^ |
| c.366C>T | 37.00 | 21.35 | 24.78 | 9.24x10^-9^ | 3.83x10^-6^ | 6.44x10^-7^ |
| c.369A>G | 29.00 | 16.74 | 20.80 | 5.04x10^-7^ | 4.28x10^-5^ | 5.11x10^-6^ |
| c.585delG | 45.45 | 27.26 | 27.28 | 1.34x10^-10^ | 1.78x10^-7^ | 1.76x10^-7^ |
