# Supplementary material for: Two-Exon Skipping within MLPH Is Associated with Coat Color Dilution in Rabbits
Source: PLoS One. 2013 Dec 20;8(12):e84525. doi: 10.1371/journal.pone.0084525 (PMC3869861; doi:10.1371/journal.pone.0084525)
Supplement: Table S3 — Distribution of the melanophilin (MLPH) c.111-5CγA, c.585delG and c.953T>C mutations in rabbits of different breeds. The dilution status and genotype with corresponding number of animals and SNP genotypes are given. Bold faced letters indicate the dilute individuals, in which SNP genotypes differ between c.111-5C>A and c.585delG. The two individuals with less obvious dilution status are given at the bottom of the table. Their dilution status is indicated as “dark-dilute” (dd*). (DOC) [file pone.0084525.s006.doc]

**Table S3.** **Distribution of the melanophilin (*MLPH*) c.111-5C>A, c.585delG and c.953T>C mutations in rabbits of different breeds.** The dilution status and genotype with corresponding number of animals and SNP genotypes are given. Bold faced letters indicate the dilute individuals, in which SNP genotypes differ between c.111-5C>A and c.585delG. The two individuals with less obvious dilution status are given at the bottom of the table. Their dilution status is indicated as “dark-dilute” (dd*).

| Rabbit  breed | Fur  color | Dilute  genotype | Number  of animals | SNP c.111-5C>A | | | SNP c.585delG | | | SNP c.953T>C | | |
| --- | --- | --- | --- | --- | --- | --- | --- | --- | --- | --- | --- | --- |
|  |  |  |  | C/C | A/C | A/A | w/w | w/del | del/del | T/T | C/T | C/C |
| Netherland  Dwarf | Wild Type | D- | 11 |  | x |  |  | x |  |  | x |  |
|  | Wild Type | D- | 2 |  | x |  | x |  |  |  | x |  |
|  | Wild Type | D- | 1 | x |  |  | x |  |  | x |  |  |
|  | Wild Type | D- | 1 | x |  |  | x |  |  |  | x |  |
|  | Dilute | dd | 4 |  |  | x |  |  | x |  |  | x |
|  | Dilute | dd | 1 |  |  | x |  |  | x |  | x |  |
| Lionhead  Dwarf | Wild Type | D- | 1 |  | x |  |  | x |  |  | x |  |
|  | Wild Type | D- | 1 |  | x |  | x |  |  | x |  |  |
|  | Wild Type | D- | 1 |  | x |  | x |  |  |  | x |  |
| Loh | Wild Type | D- | 2 | x |  |  | x |  |  | x |  |  |
| Netherland  Dwarf x Loh | Wild Type | D- | 5 |  | x |  |  | x |  |  | x |  |
|  | Wild type | Dd | 5 |  | x |  |  | x |  |  | x |  |
|  | Dilute | dd | 4 |  |  | x |  |  | x |  |  | x |
| Vienna Blue | Dilute | dd | 2 |  |  | x |  |  | x |  |  | x |
| Dwarf Lop | Wild Type | D- | 2 | x |  |  | x |  |  | x |  |  |
|  | Wild Type | D- | 1 |  | x |  |  | x |  |  | x |  |
|  | Wild Type | D- | 1 |  | x |  |  | x |  |  |  | x |
|  | Dilute | dd | 1 |  |  | x |  |  | x |  |  | x |
| Giant Lop | Wild Type | D- | 1 | x |  |  | x |  |  | x |  |  |
| Checkered Giant | Wild Type | D- | 1 |  | x |  | x |  |  |  | x |  |
| Rex | Wild Type | D- | 1 |  | x |  |  | x |  |  |  | x |
| Angora | **Dilute** | **dd** | **1** |  |  | **x** | **x** |  |  |  |  | x |
| Lionhead Dwarf | **Dark-dilute** | **dd*** | **1** |  | **x** |  |  |  | **x** |  | x |  |
| Loh | **Dark-dilute** | **dd*** | **1** |  |  | **x** |  | **x** |  |  |  | **x** |
