# Supplementary material for: Two-Exon Skipping within MLPH Is Associated with Coat Color Dilution in Rabbits
Source: PLoS One. 2013 Dec 20;8(12):e84525. doi: 10.1371/journal.pone.0084525 (PMC3869861; doi:10.1371/journal.pone.0084525)
Supplement: Table S4 — Primer sequences with their product sizes (P) for amplification of sequences within melanophilin (MLPH) to be used for sequencing, long range PCR or genotyping as well as the targeted region and the appropriate template. The primers were designed to work at an annealing temperature of 60°C. In the case of performing a mismatch PCR for digestion, the base pairs, which were changed within a primer relative to the reference sequence, are highlighted in gray. If the product of a pair of primers was enzymatically digested for genotyping, the enzyme used is given. (DOC) [file pone.0084525.s007.doc]

**Table S4. Primer sequences with their product sizes (P) for amplification of sequences within melanophilin (*MLPH*) to be used for sequencing, long range PCR or genotyping as well as the targeted region and the appropriate template.** The primers were designed to work at an annealing temperature of 60°C. In the case of performing a mismatch PCR for digestion, the base pairs, which were changed within a primer relative to the reference sequence, are highlighted in gray. If the product of a pair of primers was enzymatically digested for genotyping, the enzyme used is given.

| Purpose | Target | Template | Primer F (5’->3’) | Primer R (5’->3’) | P | Enzyme |
| --- | --- | --- | --- | --- | --- | --- |
| Sequencing | exon 1 to exon 2 | cDNA | GCTTAGCGACCTCCACAGG | CTCCTGAGGTCGAAGTCTCG | 140 | - |
|  | exon 2 to exon 6 | cDNA | ACCCTGCCCAGAAGCAGA | ATGGAGAGGAGCCGCTTT | 588 | - |
|  | exon 5 to exon 9 | cDNA | ATGAGCATGGAGAGCCAGAC | CTCCTCCAGCCTTTTCCTG | 584 | - |
|  | exon 8 to 3’UTR | cDNA | GCTGGACCTCGTCTGACAGT | GAGAGCAGACAGCGAGAGC | 770 | - |
|  | intron 2 to exon 4 | gDNA | AAGCCTGGTGGAGCCAAG | TTCGTAGTACCACTCGAGAGAGC | 402 | - |
|  | exon 3 to intron 4 | gDNA | ATTCAGAGGGAGAGCTCCAAG | AGACAGGCATGCACTCACAC | 503 | - |
| Long Range PCR | exon 3 to exon 5 | gDNA | ATTCAGAGGGAGAGCTCCAAG | GTCGCTGTCTCCACTTCTCTCCTC | 5026 | - |
| Genotyping | c.1-10A>G | gDNA | GGCCCTGCTGTTTCAGGTGTGCCCCTGCC | CTCCTGAGGTCGAAGTCTCG | 129 | BslI |
|  | c.1-1G>A | gDNA | GGCCCTGCTGTTTCAGGTGTGCCCCTGCC | CTCCTGAGGTCGAAGTCTCG | 129 | BccI |
|  | c.111-5C>A | gDNA | AAGCCTGGTGGAGCCAAG | CACTGCCTTCTGCTGTTGAC | 183 | HphI |
|  | c.214C>T | gDNA | ATTCAGAGGGAGAGCTCCAAG | TTCGTAGTACCACTCGAGAGAGC | 335 | BslI |
|  | c.215A>G | gDNA | CACCTGAACGAGACGCACT | TTCGTAGTACCACTCGAGAGAGC | 290 | BglI |
|  | c.262A>G | gDNA | GAAGGCAGTGCCTGCACTGCGGCCTGGTC | TTCGTAGTACCACTCGAGAGAGC | 229 | BstEII |
|  | c.366C>T | gDNA | GGTGAAGATGGGCTCTCTCGAGTGGTCATA | AGACAGGCATGCACTCACAC | 202 | NdeI |
|  | c.369A>G | gDNA | CCCGTGTCTCTGCTCAGG | tgccgaatcgcttgaagcggggccgcacgtg | 137 | MwoI |
|  | c.585delG | gDNA | GTCTGGTCCTGTCCTTCGAG | GGTCTGAACGCCACTGCT | 246 | SmlI |
